# New insights into maladaptive vascular responses to donor specific HLA antibodies in organ transplantation

**DOI:** 10.3389/frtra.2023.1146040

**Published:** 2023-04-28

**Authors:** Adriana Franco-Acevedo, Johanna Comes, Julia J. Mack, Nicole M. Valenzuela

**Affiliations:** ^1^ Department of Pathology and Laboratory Medicine, University of California, Los Angeles, CA, United States; ^2^ Department of Medical Biochemistry, Academic Medical Center, University of Amsterdam, Amsterdam, Netherlands; ^3^ Department of Medicine, Division of Cardiology, University of California, Los Angeles, CA, United States

**Keywords:** endothelial, HLA antibodies and/or donor-specific antibodies, endothelial to mesenchymal transition (EndoMT), vasculopathy, transplant, antibody-mediated rejection

## Abstract

Transplant vasculopathy (TV) causes thickening of donor blood vessels in transplanted organs, and is a significant cause of graft loss and mortality in allograft recipients. It is known that patients with repeated acute rejection and/or donor specific antibodies are predisposed to TV. Nevertheless, the exact molecular mechanisms by which alloimmune injury culminates in this disease have not been fully delineated. As a result of this incomplete knowledge, there is currently a lack of effective therapies for this disease. The immediate intracellular signaling and the acute effects elicited by anti-donor HLA antibodies are well-described and continuing to be revealed in deeper detail. Further, advances in rejection diagnostics, including intragraft gene expression, provide clues to the inflammatory changes within allografts. However, mechanisms linking these events with long-term outcomes, particularly the maladaptive vascular remodeling seen in transplant vasculopathy, are still being delineated. New evidence demonstrates alterations in non-coding RNA profiles and the occurrence of endothelial to mesenchymal transition (EndMT) during acute antibody-mediated graft injury. EndMT is also readily apparent in numerous settings of non-transplant intimal hyperplasia, and lessons can be learned from advances in those fields. This review will provide an update on these recent developments and remaining questions in our understanding of HLA antibody-induced vascular damage, framed within a broader consideration of manifestations and implications across transplanted organ types.

## Introduction

Transplantation is an important treatment for end-stage organ failure. In 2022, the United Network for Organ Sharing (UNOS) reported its one millionth transplant, and in the United States there are more than 350,000 people living with an organ transplant. Despite its success stemming from improvements in surgical techniques and advances in immunosuppression and histocompatibility, median graft survival continues to lag behind recipient life expectancy. Within one year of transplant, generally 90% or more of grafts are still functioning, but acute rejection rates and 5 year graft survival rates are highly disparate across organs (ranging from 49.2% to better than 85%). These are summarized from OPTN and SRTR data in Table 1.

**Table 1:** Transplant waitlist and rejection rates across organs in the US.

Organ	Number of candidates on waitlist until Feb. 2023	Number of waitlist additions in 2022	Number of transplants performed in 2022	Acute rejection <1 year (adult) % of patients	Graft survival at 1 year (primary transplants)	Graft Survival at 5 years (primary transplants)
All	104,004	62,592	42,888	–	–	–
Kidney	88,722	42,232	25,499	6.8	94.7	78.6
Liver	10,602	13,180	9,528	11.5	89.6	72.8
Heart	3,350	5,023	4,111	23.6	90.5	77.7
Kidney/Pancreas	1,917	1,443	810	12.5	95.8	81.4
Lung	955	3,104	2,692	14.6	87.2	53.5
Pancreas	844	399	108	21.8	81.8	60.1
Intestine	211	5,023	82	41.2	77.2	50.6
*Source*	*OPTN national data* (1)	*OPTN national data* (1)	*OPTN national data* (1)	*OPTN/SRTR Annual Report 2020* (2)	*OPTN National data* (1)	*OPTN national data* (1)

The long-term success of solid organ transplantation is hampered by the frequent incidence of acute rejection and development of transplant vasculopathy (TV). Chronic rejection culminating in transplant vasculopathy is reported in all transplanted organs and vascularized tissue types with an incidence ranging from 20%–50% by 10 years post-transplant (3–7). It manifests as concentric inward thickening of the intimal layer of donor blood vessels. This neointimal hyperplasia is in fact a conserved maladaptive response to chronic injury, that occurs after a wide variety of diseases with different etiologies, including systemic sclerosis, STING-associated vasculopathy, sickle cell vasculopathy, and neointimal hyperplasia of grafts and arteriovenous fistulas. The majority of these diseases have an inflammatory component. Indeed, in transplantation in particular, there is a strong association between prior acute alloimmune injury and later development of transplant vasculopathy. Nonetheless, causal connections between inflammation and chronic progression of transplant vasculopathy have been lacking. As a result, the field critically needs insights that reveal new therapeutic avenues to halt or reverse vascular occlusion and fibrosis.

This review focuses on the role of vascular endothelial cells in the pathobiology of acute transplant rejection and chronic vascular proliferative disease, and discusses recent advances in clinical and experimental knowledge indicating a putative mechanistic bridge between these processes.

## Acute antibody-mediated rejection

Rejection of organ transplants is a result of recognition of donor proteins, particularly the highly polymorphic human leukocyte antigens (HLA), as foreign by the recipient immune system. Allorecognition of nonself is mediated by multiple immune compartments, including T cells, B cells/antibodies, NK cells, and, more recently appreciated, innate myeloid cells. Alloimmunity may be preformed prior to transplant, as a result of prior exposure to allogeneic tissue through pregnancy, transfusion, tissue grafting or failed organ transplant; or it may emerge post-transplant. Mainstay immunosuppression consisting of calcineurin inhibitors, anti-proliferatives and steroids, effectively prevents severe acute T cell mediated rejection (TCMR), although the incidence of early acute rejection ranges widely, from 6.82% in the kidney to 37.84% among intestinal transplant recipients [OPTN]. Additionally, AMR is a common occurrence in the first year post-transplant and beyond.

Histological antibody-mediated rejection criteria vary across organs [see our prior review (8), but AMR is generally diagnosed by the histological presence of complement deposition (C4d/C3d), mononuclear cell infiltration, and/or microvascular inflammation (9), with or without a requirement for concurrent detection of donor specific HLA antibody (DSA) by serological methods. Clinical AMR presents with organ dysfunction, while subclinical AMR can be detected on surveillance biopsy in patients with otherwise stable organ function metrics. Transcriptomic studies of allograft biopsies have universally shown signatures of endothelial, NK cell, macrophage and endothelial-associated gene expression during antibody-mediated rejection that appear to be conserved across organs (10). Among the most strongly increased genes are the CXCR3 chemokines *CXCL11* and *CXCL9*, and HLA class II genes *HLA-DRB1, -DQA1, and -DPB1*, all of which are IFNγ inducible.

### Clinical significance

Donor specific antibodies (DSA) are associated with poor prognostic in graft survival in all transplanted organs (11–14). DSA may be preformed in allosensitized individuals, or develop in the post-transplant period. In kidney transplantation, patients with DSA had a higher risk of graft loss after both living and deceased donation, and unfavorable long-term cardiac and cerebrovascular outcomes (11, 15, 16). Further, the presence of pre-transplant DSA (DSApos) was associated with ABMR-related graft failure in the first year after transplantation, unlike T cell mediated rejection (TCMR). Interestingly, the biopsies from patients with DSA were associated with graft rejection due the development of intra-arterial thrombi or thrombotic microangiopathy in the allografts (12). Furthermore, there was a negative association between delayed graft function (DGF) and DSApos that influenced 5-year graft survival prognosis (17). In a decreased-donor kidney transplant cohort, patients with DGF and DSA pre-transplant had a seven fold greater risk of graft failure (18). Pre-formed DSA in lung transplant recipients is associated with more days of mechanical ventilation and longer index hospitalization after transplantation (19).

Another factor associated with negative graft prognosis is *de novo* DSA (dsDSA) antibodies, which appear after the first three months post-transplant. Patients who develop dnDSA have more mismatches for HLA-A, B, and DR (11). New approaches have established a negative prognostic relationship between more eplet mismatches in HLA-DQ in kidney transplants, that are associated with *de novo* DSA formation, graft failure, and rejection (20, 21). Wiebe et al. reported that recipients who developed class II dnDSA alone or class I and II dnDSA had poor graft survival. The authors stratified the alloimmune risk by the HLA-DR/DQ single molecule eplet mismatch and reported that all categories are associated with dnDSA development, T cell-mediated rejection, antibody-mediated rejection, and all cause graft loss (13). This characterization at the molecular level of donor/recipient HLA mismatches could improve graft survival prognostic and immunosuppression therapy strategies (13, 14, 20). The molecular donor/recipient HLA mismatches in kidney transplantation influence the anti-donor cellular alloimmune activation affecting *de novo* humoral alloreactivity (21). In kidney transplant biopsies, DSA and HLA B eplet MM were associated with peritubular capillary C4d deposition (22). These results agree with the analysis of transplant glomerulopathy in patients with HLA-DSA, which was associated with microvascular inflammation including glomerulitis, peritubular capillaritis, C4d deposition, and interstitial inflammation. Interestingly, the patients with HLA-DSA that became negative had a similar probability of graft survival or failure as HLA-DSA negative patients (23). In heart transplant recipients, Zhang et al. established an average of 2000 days for the appearance of dnDSA, especially against HLA-A and HLA-B antigens, compared with later development of HLA-DR or DQ. Furthermore, patients with HLA-DQ dnDSA had lower survival. This result is consistent with kidney and liver transplant studies that postulate the HLA-DQ mismatch is more immunogenic than the other loci (14, 24). Another study reported that dn-DSApos patients have around three times more risk of graft loss or chronic rejection compared to dn-DSAneg patients. Moreover, the higher risk of rejection due to ds-DSApos was significant for the pediatric population. The analysis of allograft rejection alone, demonstrated around 6 times higher rejection rate in the long-term follow up for the patients with dn-DSApos (25). The report of liver biopsies from pediatric recipients underlines an association between DSA and inflammation due the prevalence of C1q in inflammation and fibrosis, and higher number of lymphoid cells in the portal area (26).

Thus, there is substantial evidence of the negative association of HLA-DSA and *de novo* DSA in the diverse types of organ transplantation. Continual monitoring of donor specific antibodies, in the context of screening of eplet mismatches, may further advance the field to avoid or reduce allograft loss.

#### Mechanisms of acute antibody-mediated rejection

**Figure 1:**
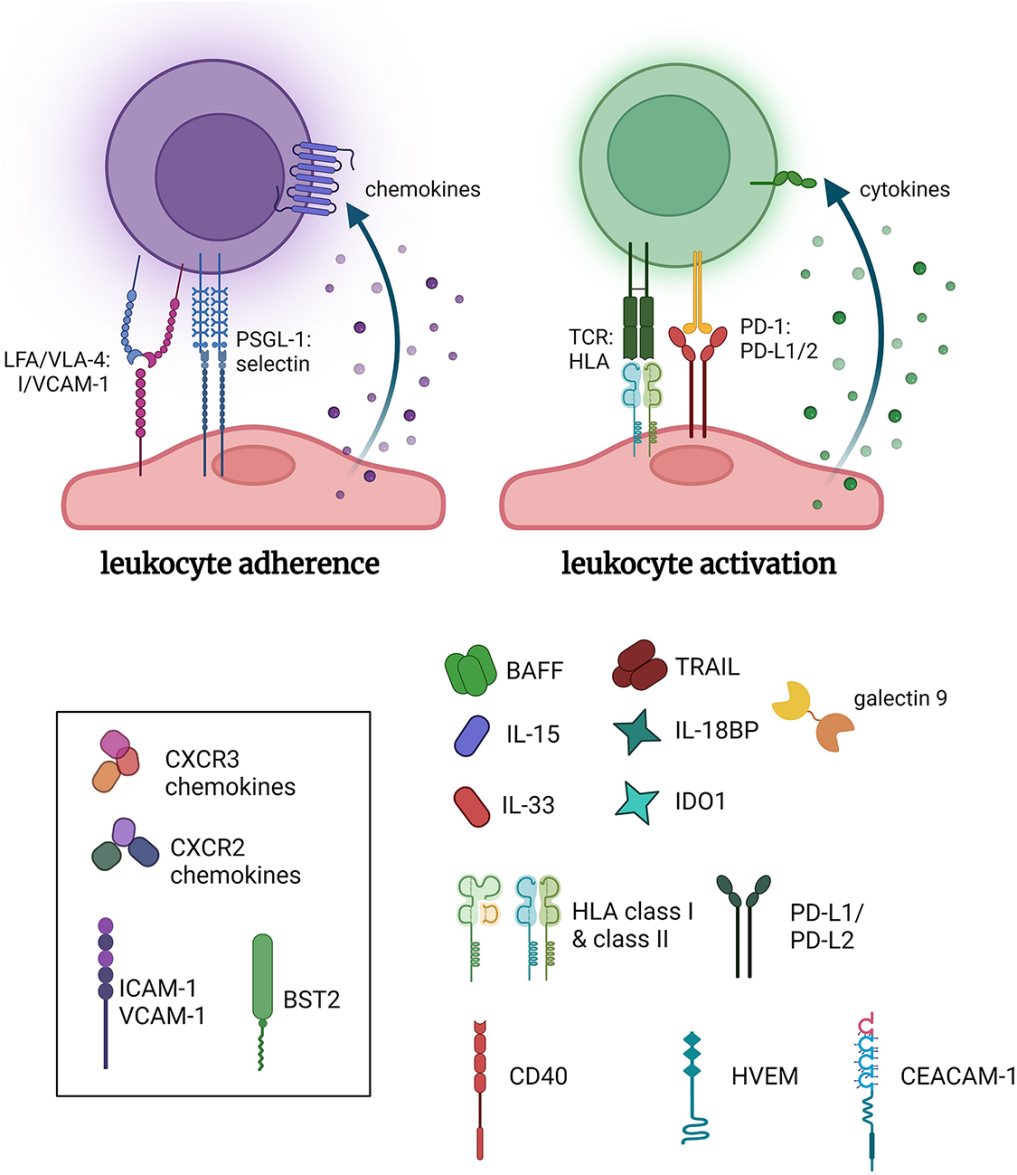
Pro-inflammatory functions of endothelial cells. Endothelial cells inducibly express a host of adhesion molecules and chemokines that promote leukocyte recruitment. Additionally, select stimuli, such as IFNγ, cause conditional expression of HLA molecules including HLA class II, and several costimulatory molecules and cytokines, which collectively can influence the activation of allogeneic leukocytes. *Made in biorender*.

**Figure 2:**
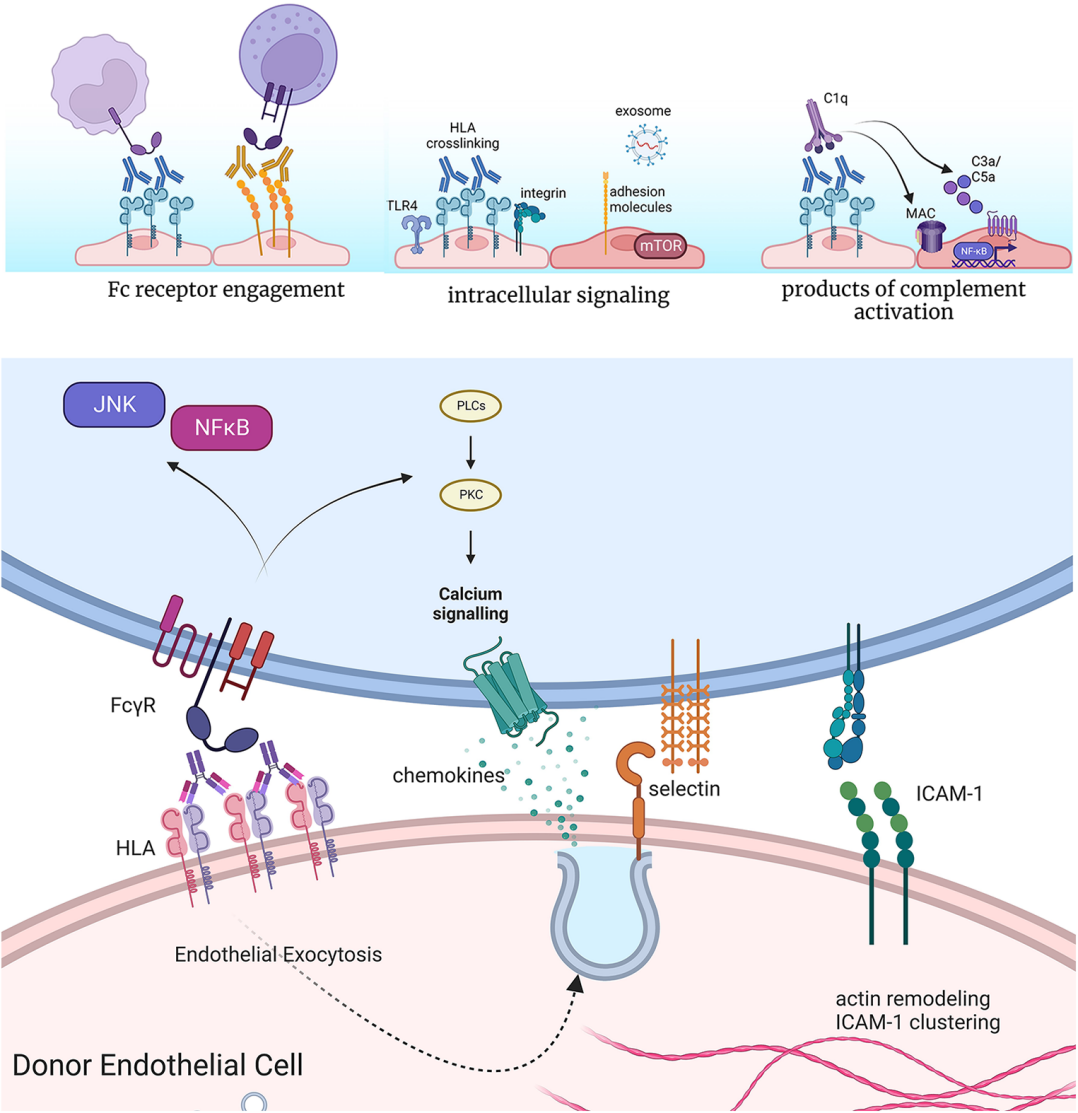
Effects of anti-HLA antibodies on acute inflammatory phenotypes of endothelium. Antibodies bound to the surface of endothelial cells can engage Fc gamma receptors on NK cells, neutrophils, and monocytes to enhance their adhesion and activation. Crosslinking of HLA by antibodies causes complex formation with TLR4 and integrin molecules at the cell surface of endothelium, which promotes intracellular signaling. This results in increased activation of pro-growth and survival signaling, predominantly through mTOR, as well as expression of adhesion molecules and release of exosomes. Finally, high levels of HLA antibodies can activate the classical complement cascade, producing split products C3a and C5a, which in turn can act on endothelial cells through their G protein coupled receptors for these molecules. In cases of extensive complement activation, deposition of the membrane attack complex (MAC) causes intracellular activation of NFκB, promoting inflammatory gene expression. Endothelial cells rapidly exocytose Weibel-palade body vesicles to externalize P-selectin, through which neutrophils, platelets and monocytes can tether. Cytoskeletal remodeling also promotes clustering of pre-formed ICAM-1 molecules, which supports tethering and firm adhesion of adhering leukocytes. Concurrent engagement of Fc receptors further enhances the strength of the adhesion. *Made in biorender*.

Decades of research have elucidated the mechanisms that underpin antibody-mediated rejection, which has been reviewed extensively elsewhere (27). Endothelial cells are highly and specifically responsive to a wide array of cytokines, microbial products, and damage-associated signals. They are capable of selectively upregulating adhesion molecules, chemokines, and antigen presentation molecules that are specific to the stimulus, and which exert an important influence on the innate and adaptive immune compartment beyond simple recruitment (Figure 1). DSA in particular provide multiple independent signals that exert effects on many graft cell types (28–34) (Figure 2). Here we will briefly review the early effects on vascular endothelial cells with a perspective of the temporal dynamics and relevance to long-term vascular changes.

DSA binding at the lumenal donor endothelial cell surface can trigger activation of the classical complement cascade, yielding upstream products C3a and C5a within an hour of exposure (35, 36). C5a in particular is pro-inflammatory for endothelial cells, which rapidly release the adhesion molecule P-selectin (35, 36), and later, after 2–4 h, transcriptionally upregulate a host of pro-inflammatory chemokines and adhesion molecules (37). Extensive complement activation can also produce the membrane attack complex (MAC) that damages cell membrane integrity, although lytic deposition is now rare beyond the context of hyperacute rejection. One explanation for their relative resistance to complement-induced lysis is that endothelium expresses high levels of complement regulatory proteins, including DAF, CD55/CD59, and complement factor H. Additionally, low levels of antibody-induced complement deposition elicit survival signaling through Akt/BCL2/BCL-XL in endothelium (38, 39) to antagonize cell death.

Nonetheless, sublytic deposition of complement promotes pro-inflammatory endothelial cell signaling. The Platt lab first demonstrated that pore formation by MAC induces IL-1α and tissue factor production by endothelium (40). The Pober and Jane-Wit groups went on to describe the subsequent activation by sublytic MAC deposition of non-canonical NFκB/NIK and IL-1β/IL-18 pathways. These signaling pathways converge to promote early expression of adhesion molecules and chemokines after 3–4 h (41–43).

In addition to complement activation, bivalent antibodies binding to HLA molecules can force crosslinking of these molecules and activate several parallel intracellular signaling events. It is hypothesized that this signaling is an upshot of endogenous mechanisms of outside-in engagement of HLA by TCR at the interface of antigen presenting cells and T cells (44).

Most of the literature has described the effects of antibodies against HLA class I molecules. Within minutes, phosphorylation of MAPK and mTOR proteins can be detected *in vitro*. In particular, mTOR mediates phosphorylation of downstream signaling molecules S6K and S6RP, and detection of these proteins in clinical biopsies discriminates AMR from no rejection (45, 46). Further, rapid phosphorylation of Src kinases and nuclear localization of the transcription factor YAP occurs upon HLA I crosslinking (47). Next, within minutes to hours, DSA causes cytoskeletal rearrangement, including stress fiber formation and clustering of the constitutively expressed adhesion molecule ICAM-1 (48). Clustering of ICAM-1 permits the endothelial cell to more effectively support adherence of tethering leukocytes. Further, by 30 min, endothelial cells exocytose stored vesicles called Weibel-Palade bodies to externalize the adhesion molecule P-selectin and the thrombotic mediator von Willebrand Factor. Together, these events rapidly increase adhesion of platelets, monocytes and neutrophils (27, 49, 50). Lastly, over several days, endothelial cell proliferation and migratory capacity is increased, in a manner that is dependent on mTOR (51).

How does HLA crosslinking by antibodies lead to such broad cellular signaling? Work from the Reed lab demonstrated that HLA class I molecules form a complex with the endothelial integrin subunit integrin β4 (52). Thereby, HLA class I molecules evoke integrin-mediated signaling to act on the endothelial cytoskeleton and promote proliferation. Additionally, HLA class I molecules separately bind to TLR4, to trigger exocytosis of Weibel-Palade bodies, externalization of P-selectin, and adherence of monocytes (53). Whether integrin β4 is required for pro-inflammatory gene expression, or if HLA class II molecules similarly complex with cell surface receptors to elicit signaling, remain open and important questions.

Because HLA class II expression by endothelium is conditional *in vivo*, and lost *in vitro*, mechanistic investigation of the pathogenic effects of HLA class II antibodies has been much more limited. Initially, studies relied on IFNγ cytokine priming of endothelial cells *in vitro* to upregulate HLA class II. More recently, several groups have used HLA class II overexpression approaches to investigate the functional effects of anti-HLA II antibodies. Like HLA class I-mediated pathways, HLA class II antibodies activated Akt, mTOR and MAPK pathways (51, 54, 55), leading to increased proliferation and migration. Yet some features of HLA class II-stimulated pathways were distinct from those of HLA class I. In particular, HLA class II-mediated pathways appear to be more dominated by ERK (51, 55). This signaling pathway divergence has possible implications for therapeutic targeting of chronic antibody-induced injury, depending on the HLA target class of DSA.

### Endothelial release of extracellular exosomes

**Figure 3:**
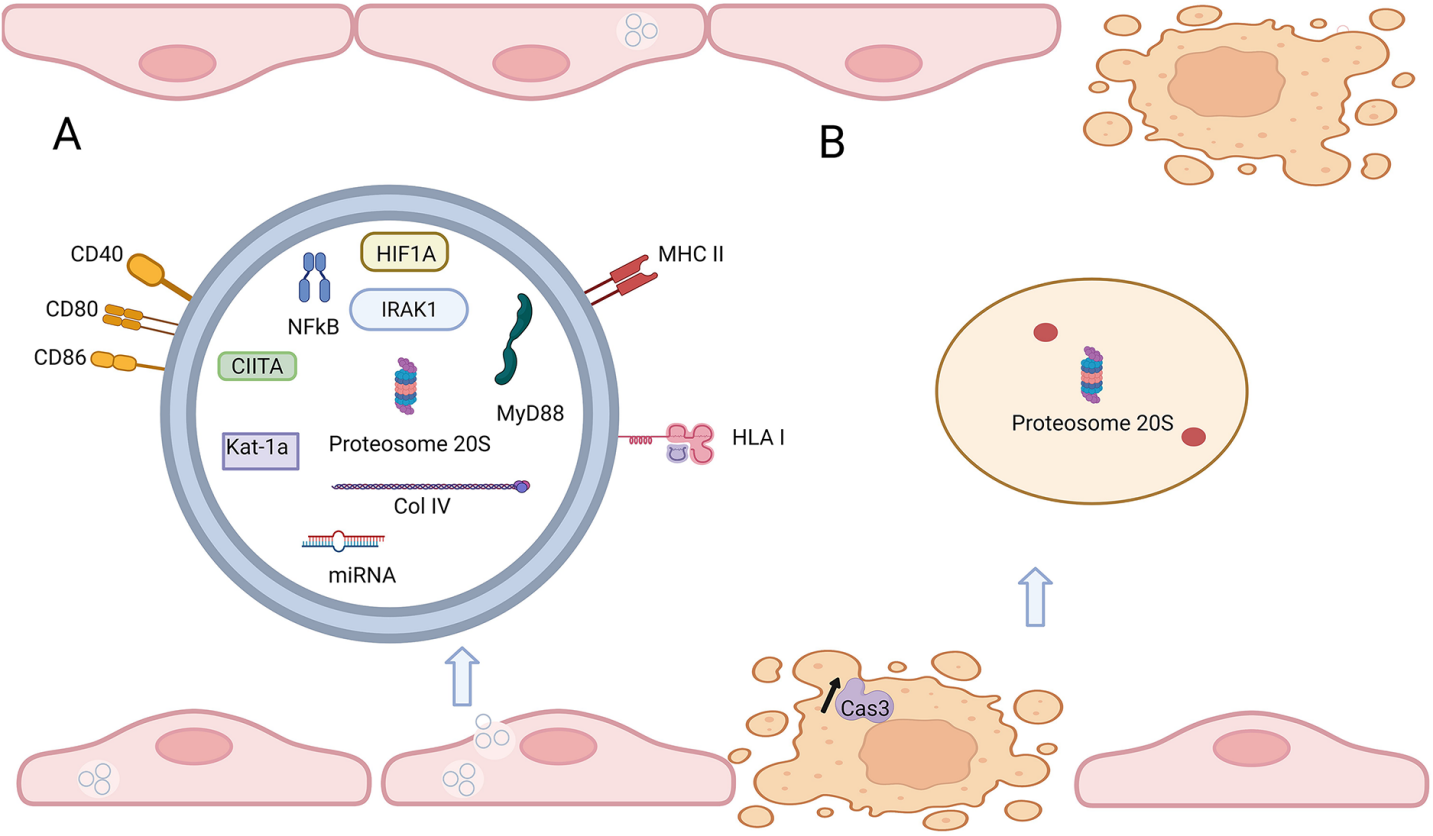
Exosome and exosome-like vesicles (ApoExo) released during graft rejection. **(A)** Molecules present or overexpressed in exosomes from patients undergoing lung transplant rejection, compared to stable transplants. **(B)** ApoExo released after Caspase 3-dependent death of microvascular endothelial cells. *Made in biorender*.

The role of exosomes in graft rejection has recently attracted attention. Exosomes are tissue-specific extracellular microvesicles released by many cell types, including transplanted organ cells (56). The Mohanakunar lab has investigated exosomes in lung transplant rejection, often diagnosed as bronchiolitis obliterans syndrome (BOS). They characterized circulating exosomes from lung transplant recipients undergoing chronic rejection and compared them to stable lung transplant recipients. Interestingly, not only mismatched donor HLA and self antigens were present in exosomes from patient undergoing graft rejection, but also microRNAs known to activate antibody-mediated rejection, endothelial activation, and inflammation (57). Moreover, after further investigation, they demonstrated that circulating exosomes from patients with chronic rejection after lung transplant had significantly different levels of several molecules implicated in graft rejection compared with exosomes isolated from stable lung transplant recipients. First, the costimulatory molecules CD40, CD80, and CD86 were increased, which respectively promote endothelial cells adhesion to facilitate activation of immune cell and regulate acute vascular rejection (58, 59). Additionally, transcription factors NFκB and HIF-1α were also higher. HIF-1α is a transcription factor sensitive to dioxygen, and its induction significantly improves vascular damage after transplantation in murine models and porcine kidney transplantation as summarized here (60). NFκB serves as key mediator of inflammation, mainly as an activator of pro-inflammatory gene expression (61). TLR4 pathway-associated signaling mediators IRAK1 and MyD88 are known to induce activation of NFκB (62), and were present in the exosome from transplanted patients with BOS but not in stable patients (57). Further, exosomes from patients undergoing chronic rejection also contained MHC-II transactivator (CIITA), MHC-II molecules, and 20S proteasomes (63). MHC II transactivator and MHC molecules play a critical role in acute rejection, as their role is to present processed antigen to CD4+ T cells (64). The 20S proteasome core has been shown to induce autoantibody production and accelerate rejection (65) (Figure 3A).

Profiling exosomes from patients with graft rejection compared to stable patients demonstrated that molecules contained in exosome reflect the biological state of the cells surrounding it (66). Here, the exomes harbor molecules linked to rejection and autoimmune response after organ transplant and appear to be a reliable predictive marker. Migneault et al. showed the presence of specific apoptotic exosome-like vesicles released after the Caspase 3 dependent death of microvascular endothelial cell (ApoExo). These vesicles induced pro-inflammatory responses in transplanted mice and increased the production of autoantibodies (67). Dieudé et al. confirmed that ApoExo vesicles stimulate autoantibody production using injection of apoptotic exosome-like in mice. Those injections led to increased graft rejection after transplant. After further analysis, they showed that the Apo Exo contains the 20S proteasome involved in graft rejection, and inhibition accordingly decreased immunogenicity. Circulating ApoExo and increased anti-autoantibody titers were also observed in mouse models of ischemia-reperfusion injury (65) (Figure 3B). Circulating donor exosome profiling might therefore enable a noninvasive detection of antibody mediated rejection. Going in this direction, the Vallabhajosyula group aimed to use C4d+ exosomes as predictive biomarkers to detect rejection after heart transplantation. After analyzing the plasma of four patients, they showed that only the patients that undergone AMR had exosomes containing Cd4. This finding might open a window to less invasive diagnostic methods, although the standard remains endomyocardial biopsy. In conclusion, exosomes are a promising noninvasive marker of AMR and endothelial damage as they reflect the state of the cells surrounding it. Early detection of AMR will allow for better control of vascular damage (56) and follow-up studies with larger cohorts are required.

### Endothelial immunogenicity

In addition to the well-characterized function promoting leukocyte adherence and transmigration, endothelial cells can act as semi-professional antigen presenters to enhance activation and skewing of T cells (68, 69). Although they lack B7 ligands needed to activate naïve T cells, we and others have shown that endothelium is capable of producing a wide array of pro-adhesive, antigenic and immunogenic molecules that can activate memory T cells (68–72), particularly in response to IFNγ. Therefore, EC are able to influence alloimmune activation and tolerance.

Antibody-stimulated endothelium has direct effects on phenotypic skewing of the immune compartment. Allogeneic regulatory T cells were expanded by direct contact with HLA II antibody-treated endothelium (73), through PD-L1 (74), although as noted above, studies investigating HLA II may be confounded by pretreatment of endothelium with IFNγ, which is a strong inducer of PD ligand expression on its own. HLA class II antibody-stimulated endothelium also produced IL-6 after 48 h (55, 73). The Mooney group has carefully shown that coculture of HLA antibody-stimulated endothelial cells with allogeneic T cells promoted expansion of pro-inflammatory Th17 CD4 cells in an IL-6-dependent manner (55, 74). Th17-mediated immunity is increasingly recognized as an important, detrimental, pathway in organ transplantation. Interestingly, IL-17 augments B cell activation and antibody production, suggesting that there may be an amplifying axis by which endothelial cells stimulate generation of more alloantibodies [extensively reviewed in (75). Further, murine donors deficient in IL-6 had reduced intimal expansion of allogeneic aortic grafts, accompanied by a decrease in activation of alloreactive T cells (76). Together, these data support that graft-derived IL-6 influences adaptive alloimmune responses. These results are therefore intriguing in the context of IL-6 and IL-6R antagonists currently under investigation for the reduction in HLA donor specific antibodies, and prevention and treatment of AMR (NCT03380377, NCT04561986). It will be interesting to see the long-term chronic rejection outcomes in these trials.

Lastly, HLA antibodies bound to endothelium can form a bridge through the Fc gamma receptors (FcγRs) to enhance adherence of NK cells, monocytes and neutrophils (27, 77, 78). For example, we showed *in vitro* that high affinity FcγRIIa variants expressed on monocytes exhibited increased adherence to HLA antibody-treated endothelial cells with IgG1 and IgG3 subclasses (27). Further, Arnold at al (79). demonstrated that renal transplant recipients carrying high affinity FcγRIII variants (expressed on NK cells) had greater microcirculation inflammation in their biopsies, and a higher production of IFNγ when cultured with HLA DSA-coated target cells. NK cell activation through FcγRs elicits multiple pathogenic effector functions, including pro-inflammatory cytokine expression and antibody-dependent cell lysis (80, 81), although histological evidence for this is difficult to identify. Interaction with HLA antibody-exposed endothelium through FcγRs can also influence macrophage differentiation, at least *in vitro*, and promote a unique remodeling phenotype, as shown by the Reed lab (82). Markers of alternatively activated macrophages have also been detected in clinical transplant biopsies (83), supporting the potential relevance of these observations.

One major remaining gap between experimental models and clinical data is that the strong IFNγ gene expression signature in acute AMR has yet to be mechanistically linked back to the intrinsic signaling activated within the graft by DSA. IFNγ is a central cytokine in the pathogenesis of organ transplant rejection. Although it exerts complex and contradictory effects on allograft outcome, animal models have demonstrated that IFNγ production by infiltrating leukocytes (84, 85) and donor IFNγ responsiveness both contribute to acute and chronic rejection (86, 87). Rejection can be reduced by deficiency of IFNγ-stimulated effectors such as HLA class II or CXCL10 within the transplanted organ (88, 89), while intragraft PD ligands attenuate alloimmune responses (90, 91). Deletion of IFNγ receptors in the graft abrogates MHC expression and immune cell infiltration (92). IFNγ activates the transcription factors STAT1 and IRF1 to trigger expression of interferon stimulated genes (ISGs). Reflecting the importance of this pathway, antagonism of intragraft STAT1 ameliorates rejection of heart transplants in mice (93). We found that stimulation of human endothelium with HLA antibodies in the presence of complement caused secretion of IFN-related CXCR3 chemokines (42). It also seems reasonable that IFNγ-induced endothelial activation is a secondary effect of HLA antibodies on the vasculature, as a sequela of infiltration of IFNγ-producing NK cells (94–96).

Another substantial gap in knowledge is organ-specific differences in endothelial response to AMR-related injury. There are long-standing differences in the rates of rejection and failure, and perceived risk of pre-formed DSA across transplanted organ types. New single cell –omics technologies are revealing astounding fundamental organotypic features of endothelial cells across tissue types (97–99), that may provide some clues to tissue-specific alloimmune injury. As yet, the translation of this knowledge to inflammation, including in context of transplant, remains to be investigated. We initiated this question by asking if endothelium from heart, lung, liver and kidney exhibited differential responses to inflammatory cytokines and antibodies + complement (42). We found that endothelium from the liver failed to upregulate the canonical tethering adhesion molecule E-selectin, and expressed lower VCAM-1, in response to inflammatory stimuli, compared to endothelial cells from other organs. We also observed that cardiac endothelial cells upregulated VCAM-1 to a greater extent than other endothelial cell types, and that patterns of chemokine expression varied. Lastly, we identified a large number of immune-regulatory transcripts that were differentially enriched in an organ-specific manner in endothelium, pointing to an unexplored contribution of intrinsic identity in differential organ transplant outcomes.

Recent advances in the induction of alloimmune tolerance have demonstrated that protocols are effective in liver and kidney transplant recipients. These have been translated to promising clinical approaches currently under investigation. However, animal models have disappointingly revealed that heart and lung allografts are refractory to the same tolerance induction protocols (100–102). Clinical experience also demonstrates protection of more susceptible organs from rejection in multi-organ transplant patients. These observations suggest that there is organotypic heterogeneity in the mechanisms of alloimmune activation and tolerance, likely at the level of the graft parenchymal cells. It is commonly accepted that liver endothelial cells have a uniquely tolerogenic function, deriving from their scavenger functions and refractoriness to PRR ligands exposed *via* the digestive input (103). However, it has never been directly tested whether endothelial cells from other organs, especially the kidney, heart and lung, differ in their propensity and quality of T cell activation, nor how one organ may so profoundly shape the alloimmune response that it prevents rejection of another. Despite existing clues, there exists a major gap in definitive knowledge of whether heart and lung endothelial cells are intrinsically less tolerogenic than liver and kidney endothelium, particularly under inflammatory conditions. This lack of understanding hinders the application of tolerance protocols to thoracic organs.

In summary, experimental models have shown that immediate changes occur in vascular endothelial and smooth muscle cells exposed to HLA antibodies. These events are reflected in the histology of biopsies during acute antibody-mediated rejection (8). The acute responses can be exacerbated by concurrent complement activation and engagement of FcγRs on myeloid cells. Moreover, long-term exposure to DSA causes a further shift in the inflammatory phenotype, that shapes the activation response of interacting T cells and monocytes. Nonetheless, much more work is needed in area of endothelial heterogeneity to reveal inflammatory and immunogenicity mechanisms in distinct vascular beds relevant to transplant outcomes.

## Chronic antibody-mediated rejection and transplant vasculopathy

Chronic antibody-mediated rejection occurs as a result of persistent injury to the graft by DSA. In the kidney, chronic AMR is recognized as a separate entity from active acute AMR. Here, transplant glomerulopathy, necessarily unique to kidneys, is diagnosed by double layering of the glomerular basement membrane. Additionally, transplant arteriopathy is apparent from occluded blood vessels (104).

In other organs, chronic and acute AMR are not yet distinguished by criteria, but there are histological features indicative of a chronic phenotype. In heart transplants, chronic AMR can affect nearly every vascular branch, ranging from capillary rarefaction in the endomyocardium to inflammatory fibroproliferative disease in the epicardial coronary arteries that can be detected by angiogram or intravascular ultrasound (9, 105). In the lung, chronic rejection has a more complex physiological manifestation, generally termed chronic lung allograft dysfunction (CLAD). AMR is significantly associated with the risk of developing CLAD. Grafts exhibit phenotypes of bronchiolitis obliterans syndrome (BOS) and/or restrictive allograft syndrome (RAS), characterized by fibrosis, microvascular damage, and occluded bronchioles (106–108). Additionally, occlusive pulmonary arteriopathy is observed in lung transplants with chronic rejection (109, 110). Diagnosis of AMR has lagged behind in the liver because of the unique resistance of this organ to overt damage by DSA. Liver transplant patients with DSA had a greater rate of development of cirrhosis (111). And, both hepatic arteries and portal veins in transplanted livers can exhibit intimal expansion accompanied by fibrosis (obliterative arteriopathy and portal venopathy, respectively) (112, 113), and this has been proposed as a feature of chronic AMR in liver transplants (7).

Transplant vasculopathy is therefore a universal, insidious disease that compromises donor blood vessels while sparing the recipient vasculature. Transplant vasculopathy affects up to 50% of transplant recipients by 10 years post-transplant and is a significant cause of graft failure and patient death. Generally, transplant vasculopathy arises from neointimal thickening of the lumenal layer by loose connective tissue and smooth muscle-like cells, leading to occlusion of arteries and veins. In the donor heart, however, larger arteries, not samples by endomyocardial biopsies, are more commonly affected. There, intimal hyperplasia with loose connective tissue and smooth muscle-like intimal cells, as well as inflammation, myocardial fibrosis and capillary rarefaction are seen in hearts with vasculopathy (105, 114–116). Beyond the affected larger vessels, in grafts with chronic rejection, other vascular beds exhibit inflammation, myocardial capillary rarefaction and fibrosis (105, 114–117). Extensive collagen deposition can be found (33). Further, many cells within the neointimal layer stain positively for αSMA in both mouse and human studies (33), leading some investigators to propose a myofibroblast or smooth muscle origin of neointimal cells. Nevertheless, the medial layer is usually intact, although some proliferating SMC can be found in the tunica media (33).

### Immunologic risk factors and predictive models

TV is chiefly thought to arise from an unresolved, chronic repair response to alloimmune-mediated injury, modified by nonimmune factors. Both recipient formation of donor specific HLA antibodies (**DSA**) and prior acute rejection episodes strongly associate with the development of transplant vasculopathy (10, 118, 119). Anti-donor HLA antibodies and prior acute rejection in the first year post-transplant were independently associated with cardiac allograft progression in a large study of heart transplant recipients (3). In an international cohort, the trajectory of CAV after heart transplantation was associated with the presence of preexisting or *de novo* circulating class II anti-HLA DSA and the development of acute cellular rejection in the first years after transplantation (3). In kidney allografts, transplant glomerulopathy is significantly associated with prior ABMR episodes, and 85% of patients with TG had anti-donor HLA antibodies (5, 104). In lung, HLA DSA positivity was significantly associated with increased risk of CLAD and graft loss, especially in patients who had prior AMR diagnosis (6). Further, dnDSA was more prevalent in Chronic lung allograft dysfunction (CLAD) compared to living-donor lobar lung transplantation (LDLLT) and had worse graft prognosis (120). Liver transplant recipients are not routinely monitored for HLA DSA; however, O'Leary et al. did find a significant correlation between the presence of HLA DSA and portal venopathy and fibrosis (7).

In the last decade, the rise of artificial intelligence (AI) and data science had led to major advances in predictive medicine. In transplantation, the application of AI could improve the prediction of graft rejection apparition and optimization of immunosuppressive treatments. In a common effort, groups from Europe and US, led by the Paris transplant group, developed the first universal tool to reliably predict the risk of kidney graft loss, called IBOX. Compared to previous attempts that used parameter like eGFR, proteinuria, HLA profiling or histology individually, or used combined parameters but were restricted by small sample size, the IBOX integrates functional, histological, and HLA antibody profiling parameters, and based on data from more than 7,000 kidney recipients. Moreover, it was validated in three randomized controlled trials. Parameters directly associated with TV are used in the algorithm, including preexisting anti-HLA donor-specific antibody and cold ischemia time (121–123). Another integrative study from the Paris transplant group determined the contribution of immune and non-immune factors in CAV development, and defined 4 different trajectories of the long-term progression of cardiac allograph. Those findings could help to adapt monitoring after transplantation and help CAV management according to likelihood of a patient to belong to one of the four trajectories (1: absence of CAV, 2: mild and late onset, 3: early onset, progressive evolution, 4: early onset rapid evolution). Class II DSA are a predominant trigger of CAV in the cohort studied, a putative sign of endothelial activation (3). The predictive model's efficiency is partly related to the accuracy of the parameter it uses. Therefore, the discovery of organotypic, rejection-specific new biomarkers will allow the improvement of the model and lead to more personalized treatment. Additional efforts to discover biomarker are ongoing, and the development to donor cell free DNA (dd-cfDNA) analysis marked a new era where bleeding, organ injury and sampling error risks of the allograft biopsy might be overcome (124). Indeed, several studies demonstrated the use of dd-cfDNA to monitor heart rejection. For example Knutten et al. have shown that dd-cfDNA values in plasma were significantly associated with cardiac rejection in a cohort of 87 patients (125). These results were confirmed in a recent multicenter study (126). The advantages of using cell free DNA for detection of kidney allograft are well described in this review (127). Also, in a recent review Maldonado et al. summarized these advances in personalized medicine in solid organ transplantation (128). Yet, no cell free DNA studies have been conducted to analyze endothelial activation evolution over time, and current progress in rapid RNA sequencing and cell free DNA detection may lead to further dissection of endothelial signaling during AMR rejection.

Despite the evidence that dd-cfDNA is a peripheral marker of acute allograft injury, there are conflicting reports of the association between elevated dd-cfDNA and chronic allograft rejection. In lung transplant patients, two studies have shown that higher dd-cfDNA levels in the early post-transplant period correlated with later development of CLAD. For example, measuring dd-cfDNA in first week post-transplant among patients with PGD was significantly predictive of later development of CLAD, than the incidence of PGD alone (129). Further, higher levels of dd-cfDNA averaged over the first three months post-transplant conferred a 6.6-fold higher risk of severe CLAD (130). One small single center study did find an association after 2 years post-heart transplant, where a majority of patients with established CAV had higher levels of dd-cfDNA, compared with a minority of patients without CAV (131). In contrast, in a Spanish cohort, there was no significant difference in levels of dd-cfDNA comparing heart transplant patients with and without CAV on concurrent angiogram >1 year after transplant (132). Therefore, the utility of dd-cfDNA to indicate chronic vascular changes within the graft warrants more investigation.

### Transplant vasculopathy: mechanistic knowledge

Multiple steps contributing to CAV have now been defined. An anti-donor immune response develops which induces significant changes in vascular endothelial cells. This eventually leads to vascular occlusion and graft failure. However, clear evidence linking these events has not yet been provided. Furthermore, intermediate signals in the development of CAV have not been identified. However, the casual links filling the gap between early alloimmune injury with the progressive vascular remodeling in CAV are still mostly unidentified. Consequently, to date there are no therapies that can halt or reverse CAV.

Historically, animal models of alloantibody-induced transplant vasculopathy have been relatively challenging. One frequently used model is the grafting of allogeneic murine arteries or veins (133). Alternatively, investigators may use major or minor histocompatibility mismatches [Bm12, minor histocompatibility mismatches], or passive transfer of cells or antibodies into immunocompromised recipients, to elicit an alloimmune response leading to TV (134). Several groups have described human arterial grafts with injection of anti-HLA I antibody (32). Such grafts develop intimal thickening after 4–5 weeks and show evidence of neointimal proliferation as marked by PCNA. The findings of these vessel grafting experiments, although elegant, should be interpreted with caution when extending to whole organ allografts, because isogeneic vessel grafts also exhibit neointimal thickening due to vascular adaptive responses to differential shear stress. Moreover, treatment with monoclonal MHC class I antibody variably prompted transplant vasculopathy in murine whole cardiac allografts (9), which has been difficult to reproduce by other laboratories.

The Fairchild and Baldwin labs reported development of a new mouse model of CAV in which CCR5^−/−^CD8^−/−^ B6 recipients of MHC mismatched heart or kidney allografts develop high titers of donor specific MHC antibody and inflammation (135, 136). They found that when recipients are treated with CD4 depletion to slow DSA development and prevent T cell mediated rejection, grafts go on to develop fibrosis and CAV (137). This recapitulates the human disease, induced by chronic injury with HLA DSA, and represents a new tool with which to study the mechanisms of transplant vasculopathy.

### Endothelial to mesenchymal transition contributes to neointimal hyperplasia and fibrosis

**Figure 4:**
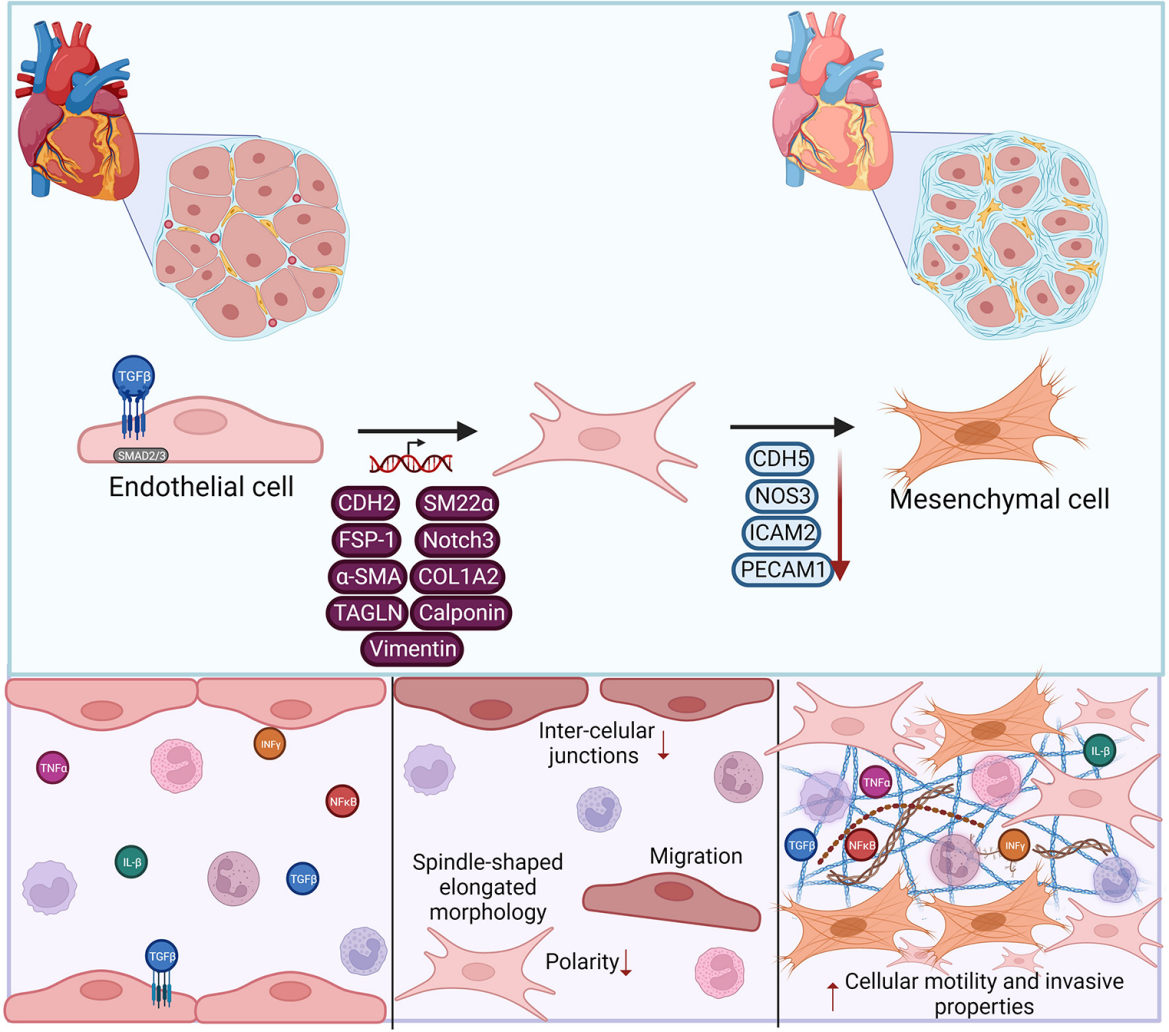
Endothelial to mesenchymal transition (EndMT) contributes to vasculopathy. EndMT development is triggered by pro-fibrotic and pro-inflammatory stimli, leading to transcriptional modifications in endothelial cells. Gene expression changes lead to phenotypic loss of canonical endothelial markers and gain of mesenchymal features. These EndMT cells can then promote extracellular matrix deposition, and expression of matrix metalloproteases, contributing to vascular remodeling. *Made in biorender*.

It is not definitively settled that lumenal endothelial cell proliferation is the definitive driving mechanism of transplant vasculopathy, and conflicting evidence exists on the contribution of medial smooth muscle cells to neointimal cellularity. Rather, neointimal cells exhibit a myofibroblast-like phenotype, which some have argued supports a smooth muscle cell origin instead. Recent studies provide convincing alternative evidence that neointimal cells do derive, at least in part, from endothelium, through the process of endothelial-to-mesenchymal transition (EndMT). EndMT is a transdifferentiation of endothelial cells characterized by loss of some or all major endothelial phenotype and markers, and acquisition of fibroblast and smooth muscle cell features (Figure 4). EndMT occurs as a normal developmental process, particularly in the heart (138), but also contributes to pathogenic vascular remodeling in the adult in numerous diseases.

EndMT features can be induced by a variety of stimuli and conditions. *In vitro*, long-term exposure to TGFβ alone or in combination with cytokines causes endothelial cells to acquire mesenchymal gene expression [CDH2, TAGLN, COL1A2, α-smooth muscle actin, SM22α, FSP1, Notch3, calponin and/or vimentin] and downregulate endothelial markers [PECAM1, CDH5, ICAM2, NOS3]. TGFβ predominantly acts through SMAD transcription factors, particularly Smad2/3, with contribution from other key transcription factors including Snail, Slug, Twist and ZEB1/2 (139, 140). EndMT can contribute to pathogenic vascular remodeling in multiple ways. Compared with quiescent endothelial cells, EndMT cells lose quiescence, are less organized, more proliferative and migratory, and produce more extracellular matrix and matrix metalloproteases (141), promoting the cellularity of the neointima. They also generate factors that can stimulate fibrosis and osteogenesis by other cells (142), and importantly display a strong proinflammatory signature that can promote leukocyte recruitment (143).

In humans, EndMT is found in many diverse diseases of occlusive vasculopathies and fibrosis, including atherosclerosis (144), pulmonary arterial hypertension, vein graft failure (145), systemic sclerosis (146, 147); SAVI/interferonopathy; and Kawasaki disease. Several studies in these non-transplant vascular occlusive diseases have shown that a key feature of EndMT is the loss of pioneer endothelial transcription factors of the ETS family. For example, lung endothelium from patients with pulmonary arterial hypertension show reduced nuclear ERG (148). ERG was also notably absent in the lumenal layer overlaying human coronary artery atherosclerotic plaques (149) and in pulmonary veno-occlusive disease (150). In scleroderma-associated vasculopathy, a related ETS factor FLI1 was lost (151). It is plausible that dysregulation of the ETS family of pioneering endothelial transcription factors may therefore play a role in transplant vasculopathy.

Animal models of fibrosis and native neointimal hyperplasia have also provided some deeper insights into the dynamics and signaling involved in this process (152–154). Moonen et al. found the presence of “myo-endothelial cells” in several models of neointimal hyperplasia, further highlighting the role of EndMT (155). Murine kidney fibrosis was rescued by deficiency in the mesenchymal transcription factors TWIST and SNAIL (156). Elegant lineage tracing experiments in the mouse showed unequivocally that fibroblast-like cells within scarred native hearts were endothelial in origin and exhibited TGFβ signaling (p-Smad2, Smad3) (154). Similarly, Cooley et al. (145) employed an inducible Endotrack mouse with an isogeneic vein graft that develops hyperplastic neointima. They too clearly demonstrated a preponderance of endothelial-derived cells in the day 35 neointima. In particular, they reported a progressive reduction of PECAM1, CDH5 and CD105 in YFP+ cells between 7 and 14 days after grafting, which were fully lost by 35 days. A concomitant increase in N-cadherin, Thy1, SMA and SM22α was observed in YFP+ EC-derived cells, although few also expressed mature VSMC markers calponin or fibroblast FSP1. The TGFβ signaling pathway p-Smad2/3 along with transcription factors Slug and Twist were increased transiently; p-Smad1/5/8 up by 3 days and persistent. Accordingly, anti-TGFβ antibody reduced neointimal formation in vein grafts (145). Longitudinal profiling of single cell expression after transient cardiac ischemia similarly highlighted the temporal acquisition of mesenchymal genes by endothelium early after injury (152). Remarkably, unlike vein grafting, in this model normality was recovered day 14, suggesting that temporary EndMT may be a normal process of remediation, that fails to resolve under chronic injury in progressive diseases like transplant vasculopathy.

### Endothelial to mesenchymal transition occurs in transplant vasculopathy

Although the upstream process that cause alloimmune injury are distinct from those in native diseases discussed above, recent experimental and clinical evidence shows that EndMT is evident in end-stage organ transplant vasculopathy. Both smooth muscle and endothelial markers are detectable within the neointima of transplanted organs with TV. For example, in CAV, expression of matrix metalloproteases (MMPs) and TGFβ can be found in affected vessels (114). Likewise, transplant vasculopathy in kidneys exhibits CD31 + SMA+ double positive cells (156). TGFβ1 and its downstream WNT signaling were specifically upregulated in renal allografts with microvascular injury; and within that pathway the genes most highly associated with graft failure are markers of EndMT: ZEB2 and SMAD3 (157). And, endothelial expression of NOTCH4 was downregulated during transplant arteriosclerosis (158), again implicating EndMT.

Similar findings come from animal models of TV. In a murine chronic rejection model, markers of repair and angiogenesis were persistently upregulated in both microvasculature and coronary artery endothelial cells of the heart (159). Although the majority of intimal cells are donor in origin, the myofibroblast-like cells did not arise from the vessel medial smooth muscle cells SMC, at least in a rodent model (115, 160). Fate-mapping experiments by the Simons and other labs in murine heart transplant chronic rejection (154), murine artery transplant (133) and vein grafting (161) models demonstrated that the neointimal smooth muscle-like cells partly derive from endothelium, with a majority of neointimal endothelial cells coexpressing αSMA and Notch3. Therefore, in many diseases of neointimal hyperplasia, including in transplant vasculopathy, endothelial cell transition to a fibroblast/smooth muscle like phenotype occurs frequently within affected vessels.

### The relationship between inflammation, antibodies and endothelial to mesenchymal transition

Given the correlative relationship between acute rejection and transplant vasculopathy, what process bridges alloimmune inflammation with vascular remodeling? Although central, TGFβ alone is insufficient to promote full EndMT. Inflammation is a critical second input for full EndMT and neointimal hyperplasia. Increased levels of both TGFβ and the inflammatory cytokine TNFα were found in CAV (158), and it has long been known that IFNγ related transcripts predict TV (162, 163). A mechanistic role for IFNγ responses is supported by experimental models (164, 165). Similarly, in native coronary artery restenosis, IFNγ-related genes were strikingly upregulated in the neointima, and mice deficient in IFNGR exhibited reduced neointimal formation (166). *In vitro*, TNFα increased CDH2^+^CD31^+^ double positive EC, as well as αSMA and SNAIL expression (167). And, the combination of IL-1β and TGFβ synergistically induces EndMT through NFκB. Additionally, IFNγ downregulated the endothelial marker *CDH5*, while upregulating mesenchymal genes *ACTA2*, *CTGF*, *TGFB2*, *COL1A1*, and PAI-1, and functional features of EndMT (168). Therefore inflammatory cytokines cause features of EndMT.

Several recent key studies strongly suggest that the acute process of AMR itself may trigger an EndMT phenomenon that could drive transplant vasculopathy. For example, it is well-established that cardiac and renal biopsies with antibody-mediated rejection exhibit marked changes in gene expression (10, 96), including endothelial-specific genes. Further, in renal allografts, markers of EndMT were evident during acute antibody-mediated rejection, and were significantly associated with the presence of microvascular inflammation (2.52 & 3.5 OR), HLA DSA (2.1 OR), and later graft loss (169, 170). In particular, Xu-Dubois et al. found that fascin1, vimentin and hsp47 were increased in kidneys with AMR, whereas such staining was absent in normal kidneys and was specifically associated with peritubular capillaritis and glomerulitis. Patients whose renal grafts had a greater degree of this staining also experienced a significantly greater deterioration of function after biopsy. These findings connect acute alloimmune vascular injury with EndMT in the physiological transplant setting and warrant follow-up studies to elucidate the mechanisms at play.

Immune complexes from patients with systemic sclerosis upregulate also TGFβ and collagen production by endothelium (171), and *in situ* complement activation was associated with phosphorylated SMAD2/3 in dialysis-associated occlusive arteriopathy (172), suggesting a conserved mechanism of antibody/complement-mediated injury and EndMT. In a new MHC antibody-driven CAV mouse model, *COL1A1* and *DLL4*, both implicated in EndMT, were significantly changed in more severe CAV with higher titers of DSA (137). *In vitro*, stimulation of human glomerular endothelium with anti-HLA class I antibodies for 48 h upregulated secretion of TGFβ (173).

In addition to direct effects on endothelial cells, it is possible that infiltrating immune cells influence the pro-fibrotic milieu and contribute to neointimal expansion and EndMT. In clinical renal transplant biopsies, there was a greater number of CD68 macrophages associated with worse degree of fibrosis, and further staining for markers CD163 and CD206 demonstrated a predominance of M2-like macrophages among the infiltrates (174). Pioneering follow-up work from Dangi et al. (175) employed single cell RNA-sequencing to profile infiltrating leukocyte gene expression in mouse renal transplants, and identified a unique inflammatory macrophage population in rejecting but not tolerized grafts. Functionally, interactions between inflamed endothelium and macrophages alters expression of Notch ligands (discussed in more detail below), which in turn promotes macrophage differentiation (176). Recently, *in vitro* studies demonstrated that HLA antibody-activated endothelium promoted skewing of adherent monocytes to a unique pro-repair phenotype characterized by higher CD68, CD162, IL-10 expression, and genes involved in phagocytosis (49).

The expansion of Th17 cells by HLA antibody-activated endothelium described above may also represent one link between acute inflammation and chronic rejection. In particular, IL-17 plays an important role in fibrosis, and mice with enhanced Th17 immunity experienced accelerated rejection and transplant vasculopathy. Moreover, IL-17 has been strongly implicated in organ fibrosis in several native diseases, including atherosclerosis and renal fibrosis. It is therefore possible that HLA antibodies trigger endothelial activation, which in turn expand Th17 immunity, and that this contributes to the long-term association with fibrosis and vascular occlusion seen in chronic rejection (75, 177–179). Taken together, this body of work suggests that antibody-induced inflammation may trigger a cycle of profibrotic signaling and vascular remodeling leading to transplant vasculopathy (Figure 4).

### Non-coding RNAs in antibody-mediated rejection and transplant vasculopathy

HLA I antibodies induced a change in the endothelial profile of miRNA expression, which was more pronounced when complement was included (180). The authors were able to further validate differential expression of miRNA within human living donor renal transplant biopsies with DSA, which showed elevated Let-7C, miR-125a-5p and miR-520e (180). Franzin et al. (181) recently reported that extracellular vesicles isolated from the plasma of 14 renal transplant patients with AMR reduced expression of hallmark endothelial cell markers CD31 and VE-cadherin, while increasing vimentin and collagen expression. These EV contained several miRNA involved in regulation of fibrotic pathways, as well as complement regulation. In particular, miR-604, miR-515, miR-let-7d and miR-590 were higher. As in the study from Xu-Dubois, the authors observed significantly increased αSMA staining in kidney allografts, particularly in the glomeruli and peritubular capillaries, with AMR compared with those without rejection. miR-let-7 is likely to be a particularly important candidate, since the Simons lab separately showed (133).

In cardiac endothelial and interstitial cells, the expression changes of miR-10a, miR-31, miR-92a, and miR-155 were associated with inflammatory response during rejection. Further, serum level of miRNA was able to discriminate patients with and without heart transplant rejection (182). In particular, in heart transplant recipients, differential levels of plasma microRNAs were particularly abundant in subjects with AMR, including Let7b, miR-142-3p (183). Within cardiac biopsies, Di Francesco et al. identified diverse miRNAs related to AMR and heart allograft rejection including miR- 31-5p, -144-3p, and miR-29c-3p, -29b-3p, -199a-3p respectively (184). Also, murine cardiac transplant models have described mechanisms by which miRNAs participate in allograft impairment. For example, deletion of miR-142 and miR-146a augmented allograft survival, while miR-142 promoted tolerance. This was associated with increased peripheral regulatory T cells (Treg) (185). Further, miR-146a decreased fibrosis formation and alleviated rejection, *via* Tregs regulation through the IFN-γ/STAT1 pathway (186). Lastly, miR-21 was upregulated in murine and human allograft vasculopathy, and its *in vivo* reduced infiltrating macrophages. The authors postulated that miR-21 is a potential target to decrease inflammatory response (187).

**Figure 5:**
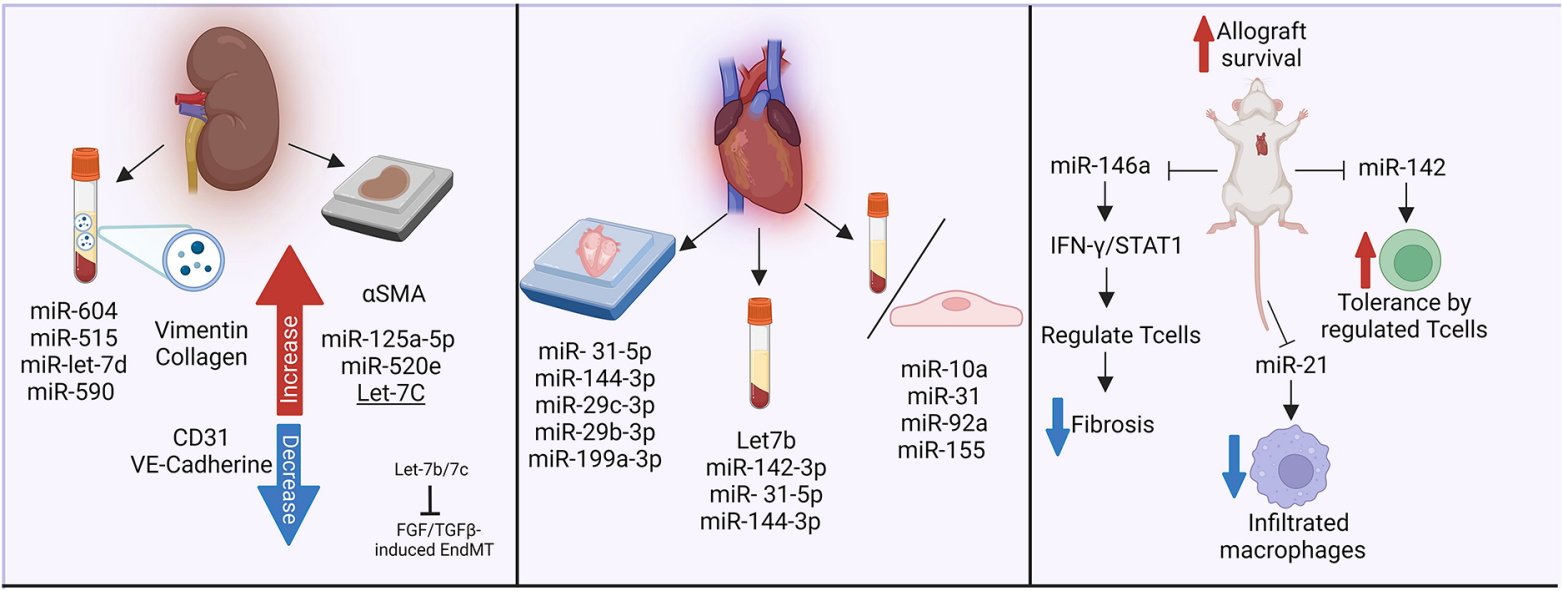
Non-coding RNAs in antibody-mediated rejection and transplant vasculopathy. The influence of miRNAs in fibrotic pathways, complement regulation, inflammatory responses and rejection has been analyzed in biopsies and plasma samples from kidney and heart transplant recipients. Murine models have further described the impact of miR-146a and miR-142 in heart transplant survival. *Made in biorender.*

Therefore, changes in non-coding RNA are observed both in experiment models and in clinical transplant rejection (Figure 5). It will be interesting to follow-up these observations with mechanistic studies to determine which are graft-derived, and their mechanistic effects on vascular changes leading to TV.

### The contribution of Notch signaling in vascular health and disease

Occurring *via* cell-cell communication, Notch ligands (Delta-like-1,3,4 & Jagged-1,2) activate Notch receptors (Notch-1,2,3,4) *via* ligand binding and subsequent cleavage events to enable Notch intracellular domain nuclear translocation and transcription *via* the RBPJ transcription factor (188). The Notch signaling pathway plays a critical role both in vascular development and blood vessel homeostasis (189). Decades of work have demonstrated the requirement of Notch signaling for effective angiogenesis, hierarchical vascular patterning, arterial specification, vessel integrity and vascular inflammation (190). Intricate control of Notch signaling is important in both homotypic and heterotypic cell-cell interactions and the loss or hyper-activation of these signaling events contribute to multiple vasculopathies, including endothelial cell activation, immune cell recruitment, vascular smooth muscle cell hyperproliferation and extracellular matrix remodeling (191). Multiple reports have demonstrated that loss of Notch activity in endothelial cells results in endothelial dysfunction, as evidenced by increased cell proliferation, loss of junctional integrity and enhanced inflammatory signaling (192). These consequences were shown to increase the atherosclerotic plaque burden in hypercholesterolemic mice lacking endothelial Notch1 compared to hypercholesterolemic control mice (193). Further evidence of its role in vascular inflammation, the presence of oxidized lipids and cytokines associated with atherosclerosis progression can suppress endothelial Notch1 expression (194). In the context of atherosclerosis, multiple Notch ligands and receptors are expressed in the atherosclerotic lesion and Notch activity in the smooth muscle compartment promotes the development of plaque cells in the fibrous cap (195). The authors also found that the sequence of Notch signaling events shared characteristics with arterial media assembly during embryogenesis and therefore point to the importance of Notch activity not only during development but also in disease states to maintain a connection between the endothelium and subendothelial smooth muscle layer (195). Furthermore, in a vein graft model, antagonism of the Notch ligand Dll4 attenuated neointimal hyperplasia, suppressed inflammatory M1 macrophage differentiation and reduced endothelial inflammatory gene expression *in vivo* (196).

Notch signaling has been reported to contribute to the pathogenesis of arteriosclerosis after transplant. Although the exact role of Notch signaling in allografted vessels and the development of transplant arteriosclerosis is still an active area of exploration, a study by Quillard et al*.* found that impaired Notch4 activity aggravated transplant arteriosclerosis by triggering endothelial cell dysfunction and apoptosis (158). Further suggesting the role of Notch signaling in vascular inflammation, the Notch family of ligands and receptors are important regulators of immune cells (197). Reports have shown that graft failure due to antibody-mediated rejection is associated with changes in Notch signaling between endothelial cells and immune cells. Specifically, endothelial Notch4 was downregulated while the Notch ligand Dll4 was upregulated in both endothelial cells and macrophages during antibody-mediated rejection (176). This endothelial cell to immune cell signaling promoted the differentiation of monocytes into macrophages with a pro-inflammatory phenotype, concurrent with production of the inflammatory cytokine IL-6. This highlights the importance of heterotypic cell interactions at the vessel wall and indicates Dll4 as a potential immunotherapy target for treatment of vascular inflammation after cardiac transplantation. With regard to vasculopathies associated with cardiac transplant, work by Norum et al*.* revealed a correlation between the Notch ligand Dll1 and cardiac allograft vasculopathy (198). The importance of Notch signaling in allograft vasculopathy is further supported by transcriptional analysis of allograft arteries showing an elevated expression of Dll4 is associated with both high titers of donor-specific antibodies and the occurrence of vasculopathy (137).

Notch signaling is also reported in the context of endothelial-to mesenchymal transition. Noseda et al*.* revealed some of the first evidence for Jagged1-Notch activation in the induction of endothelial-to-mesenchymal transition (EndMT) during cardiac development (199). The authors showed that Notch activation in endothelial cells resulted in phenotypic changes consistent with EndMT and established the idea that Notch signaling was required for proper endocardial cushion differentiation. Mounting evidence suggests that EndMT also plays a role in adult cardiovascular disease (142, 200). In fact, it was recently shown that activation of the Notch pathway can induce EndMT and promote the development of atherosclerosis (201). Staining of human atherosclerotic plaques found the co-expression of both endothelial and mesenchymal markers, thereby suggesting an intermediate stage of EndMT in the lesions (155). The researchers also showed that oscillatory shear stress promoted EndMT while high laminar shear stress suppressed this transition (155). Considering that endothelial Notch1 signaling is responsive to the level of shear stress (193), it is provoking to consider that Notch activity could be influenced by the local shear stress environment and thereby influence EndMT at the aortic wall. It is thereby possible that blood flow perturbations after engraftment could induce changes in endothelial Notch activity, thus linking the role of Notch signaling to vasculopathy after transplant.

## Translation to therapy and gaps in knowledge

Many of the *in vitro* studies described above have stimulated endothelium with monoclonal murine antibodies to HLA class I, and should be confirmed using human allele specific antibodies or alloserum. Further, experiments testing the effects of HLA II antibodies on endothelium *in vitro* are limited by the requirement to artificially induce HLA class II expression—usually through priming with IFNγ, which independently induces myriad phenotypic changes in endothelium (72). Therefore the individual effects of antibodies themselves can be difficult to discern in such studies. Some approaches have delivered HLA II genes or the master transcription factor CIITA directly to circumvent this confounder. Another important caveat is that most of the studies cited above employ a limited range of human endothelial cells, usually derived either from the umbilical cord or aorta. Extensive recent work in the vascular biology field has characterized the complex transcriptional, functional, and phenotypic heterogeneity of endothelial cells from different organs and vascular beds (97, 99, 202). Some work, including our own, has demonstrated differential responses of organotypic endothelial cells to inflammatory stimuli, including in response to HLA antibodies and complement (42, 97, 99, 202). Given disparities in outcomes across different transplant organs, it will be important for the transplantation field to extend its investigations to endothelial cells from relevant tissue-specific vascular beds. It will be particularly critical to distinguish effects on liver endothelium from others, given the unique phenotype and tolerogenic characteristics of sinusoidal endothelium.

Despite the advances summarized above, there are very few examples of how knowledge of the acute injury triggered by HLA donor specific antibodies have translated to effective therapies for CAV (Table 2). mTOR inhibitors are widely used as an alternative renal-sparing maintenance immunosuppression to calcineurin inhibitors. Several studies have demonstrated reduced neointimal thickening and CAV incidence in heart transplant patients taking mTOR inhibitors (215, 216). These clinical observations are in line with *in vitro* experiments that demonstrated a reduction in HLA antibody-induced proliferation of endothelial cells (217), which may represent one mechanism of the development of CAV. Nevertheless, mTOR inhibitors also suppress signal 3 in the activation of T cells, and this is a potential confounder in interpreting mechanism. Its independent beneficial effects on vascular changes remain to be determined.

**Table 2:** Current and novels strategies to treat chronic AMR in solid organ transplantation.

**Current treatment strategies**	
• The most validated strategy is: - Plasmapheresis, Immunoglobulin G (IVIG), Rituximab, and/ or Bortezomib (203–206) • In kidney, KDIGO recommend reducing, withdrawing, or replacing calcineurin inhibitors with mTOR inhibitors (207).	
**Novel treatment approaches**	
• Anti-Interleukin-6	
Clazakizumab • Phase three clinical trial. Kidney transplant recipients with caAMR. Decreased DSA MFI and rejection scores per biopsy (208). • Kidney transplant biopsy-proven caAMR. Patients had stable eGFR, decreased DSA and improved Treg cells in peripheral blood (209).	Tocilizumab • caAMR cohort. Patients showed stable renal function and histological injury (210). • In caAMR patients resistant to conventional treatment (IVIG + rituximab ± PLEX), Tocilizumab decreased some IgG subclasses, and anti–HLA-total (211).
• Extracorporeal photopheresis in chronic AMR patients. Reduced HLA serum levels and improved graft survival (212).	
• caAMR patients treated with BM-MSCs combined with Immunosuppressive drugs reduce DSA levels, pro-inflammatory cytokines and maintain allograft function (213).	
• Belatacept administration to caAMR patients after discontinuation of CNI decelerated renal function loss, and decrease gene expression scores associated to histological damage (214).	

mTOR, Mammalian Target of Rapamycin Inhibitors; IVIG, Immunoglobulin G; AMR, Antibody Mediated Rejection; caAMR, Chronic Active AMR; PLEX, Plasmapheresis; BM-MSCs, Bone Marrow-Derived Mesenchymal Stem Cells; CNI, Calcineurin Inhibitors.

Complement inhibition has been avidly explored as a treatment for the prevention or reversal of acute antibody-mediated rejection. Similarly, IL-6/IL-6R inhibitors are being actively investigated to reduce HLA donor specific antibodies, and may have an ancillary benefit of reducing inflammation mediated by this pleiotropic cytokine (218). Transplant vasculopathy outcomes in patients treated with IL-6/IL-6R or complement inhibitors have not been specifically reported, but are included as secondary/exploratory endpoints in several ongoing clinical trials.

## Conclusions and future directions

In summary, anti-donor HLA antibodies prompt a wide array of functional changes in allograft endothelial cells as an acute response. Emerging evidence about TGFβ production, Th17 skewing, and differential miRNA expression during acute antibody-mediated injury supports the recent separate observations that allografts with AMR and transplant vasculopathy show hallmarks of EndMT and fibrosis. Future studies linking the early processes of vascular injury with the progressive remodeling seen in transplant vessels have the potential to identify new therapeutic avenues to counteract and prevent this insidious disease.
